# TLR4 mutant mice are protected from renal fibrosis and chronic kidney disease progression

**DOI:** 10.14814/phy2.12558

**Published:** 2015-09-28

**Authors:** Ana C P Souza, Takayuki Tsuji, Irina N Baranova, Alexander V Bocharov, Kenneth J Wilkins, Jonathan M Street, Alejandro Alvarez-Prats, Xuzhen Hu, Thomas Eggerman, Peter S T Yuen, Robert A Star

**Affiliations:** 1 Renal Diagnostics and Therapeutics Unit, NIDDK, NIHBethesda, Maryland; 2 Department of Laboratory Medicine, Clinical Center, NIHBethesda, Maryland; 3 Biostatistics Program, Office of Director, NIDDK, NIHBethesda, Maryland; 4 Division of Diabetes, Endocrinology, and Metabolic Diseases, NIDDK, NIHBethesda, Maryland; 1st Department of Medicine, Hamamatsu University School of MedicineHamamatsu, Japan

**Keywords:** Albuminuria, endotoxemia, inflammasome, inflammation, spleen apoptosis

## Abstract

Chronic kidney disease (CKD) is associated with persistent low-grade inflammation and immunosuppression. In this study we tested the role of Toll-like receptor 4, the main receptor for endotoxin (LPS), in a mouse model of renal fibrosis and in a model of progressive CKD that better resembles the human disease. C3HeJ (TLR4 mutant) mice have a missense point mutation in the TLR4 gene, rendering the receptor nonfunctional. In a model of renal fibrosis after folic acid injection, TLR4 mutant mice developed less interstititial fibrosis in comparison to wild-type (WT) mice. Furthermore, 4 weeks after 5/6 nephrectomy with continuous low-dose angiotensin II infusion, C3HeOuJ (TLR4 WT) mice developed progressive CKD with albuminuria, increased serum levels of BUN and creatinine, glomerulosclerosis, and interstitial fibrosis, whereas TLR4 mutant mice were significantly protected from CKD progression. TLR4 WT mice also developed low-grade systemic inflammation, splenocyte apoptosis and increased expression of the immune inhibitory receptor PD-1 in the spleen, which were not observed in TLR4 mutant mice. In vitro, endotoxin (LPS) directly upregulated NLRP3 inflammasome expression in renal epithelial cells via TLR4. In summary, TLR4 contributes to renal fibrosis and CKD progression, at least in part, via inflammasome activation in renal epithelial cells, and may also participate in the dysregulated immune response that is associated with CKD.

## Introduction

Chronic kidney disease (CKD) leads to end-stage renal disease and death (Coresh et al. 2003), and is highly prevalent worldwide (Bello et al. 2005). CKD is associated with systemic inflammation, including increased circulating inflammatory cytokines (TNF-*α*, IL-6, IL-10) (Stenvinkel et al. 1999; Duffield 2010; Lavin-Gomez et al. 2011; Leelahavanichkul et al. 2011; Weichhart et al. 2012; Tbahriti et al. 2013). Persistent systemic inflammation contributes to CKD progression and cardiovascular events (Silverstein 2009), and is an independent risk factor for poor survival during CKD (McIntyre et al. 2011; Anders et al. 2013). Local renal inflammation and extracellular matrix deposition are crucial in the pathogenesis of tubulointerstitial fibrosis (Anders and Ryu 2011).

Chronic kidney disease is also a state of acquired immunodeficiency (Anders et al. 2013): chronic activation of immune cells by low-grade persistent inflammation causes an immunodeficient state (Descamps-Latscha et al. 1995; Minnaganti and Cunha 2001; Kato et al. 2008; Vaziri et al. 2012) often measured as lymphocyte dysfunction (Levin 2003; Naqvi and Collins 2006; Dalrymple and Go 2008; Leelahavanichkul et al. 2011). Spleen apoptosis occurs in the 5/6 nephrectomy model of CKD in mice, and pre-existing CKD worsens sepsis and sepsis-induced acute kidney injury in part due to increased serum levels of high mobility group box protein-1 (HMGB-1) (Leelahavanichkul et al. 2011), a damage-associated molecular pattern (DAMP) that is translocated to the extracellular environment by both active secretion by immune cells and passive release by necrotic or apoptotic cells (Lee et al. 2014), including apoptotic splenocytes (Leelahavanichkul et al. 2011).

While inflammation and immune dysfunction are known to participate in CKD, little is known regarding the impact of innate immunity on kidney function and CKD progression. Toll-like receptors (TLRs) are a family of receptors widely expressed that recognizes pathogen-associated molecular patterns and DAMPs, including HMGB-1. TLRs promote the activation of both immune and intrinsic renal cells in nonimmune kidney diseases (Anders et al. 2004; Lin and Tang 2014). Only TLR4 senses circulating LPS derived from gram-negative commensal bacteria in the intestine (Jin et al. 2013), and is involved with inflammation associated with other systemic chronic diseases, such as diabetes, obesity, and metabolic syndrome (Jin et al. 2013). While the role of TLR4 on kidney function has been explored for models of acute kidney injury (Wu et al. 2007; Zhang et al. 2008; Chen et al. 2011; Castoldi et al. 2012; Lu et al. 2012) and diabetic nephropathy (with TLR4 being involved on tubulointerstitial inflammation and fibrosis) (Lin et al. 2012; Liu et al. 2014), less is known regarding its impact on kidney function on models of progressive CKD that more comprehensively resemble the human disease.

To better understand the role of TLR4 on CKD progression, we first compared C3H/HeOuJ (TLR4 WT) mice with mice that have a missense point mutation in the Tlr4 gene, rendering the receptor nonfunctional (C3H/HeJ mice, henceforth called TLR4 mutant) (Hoshino et al. 1999), using a less complex model of renal fibrosis induced by folic acid. After observing that TLR4 mutant mice are protected from tubulointerstitial fibrosis we moved to a more complete model of progressive CKD. The 5/6 nephrectomy with angiotensin II infusion model better resembles human progressive CKD, with progressive rises in serum BUN and creatinine, and progressive albuminuria.

## Methods

### Animals

The National Institutes of Health (NIH) criteria for laboratory animal care were used in this study. Eight week old C3H/HeOuJ (TLR4 WT) and C3H/HeJ (TLR4 mutant) mice were purchased from Jackson Laboratory (Bar Harbor, Maine) and maintained at a NIH animal facility with free access to water and regular chow. This study was approved by the NIDDK, NIH Animal Care and Use Committee.

### Folic acid model

**Figure 1:**
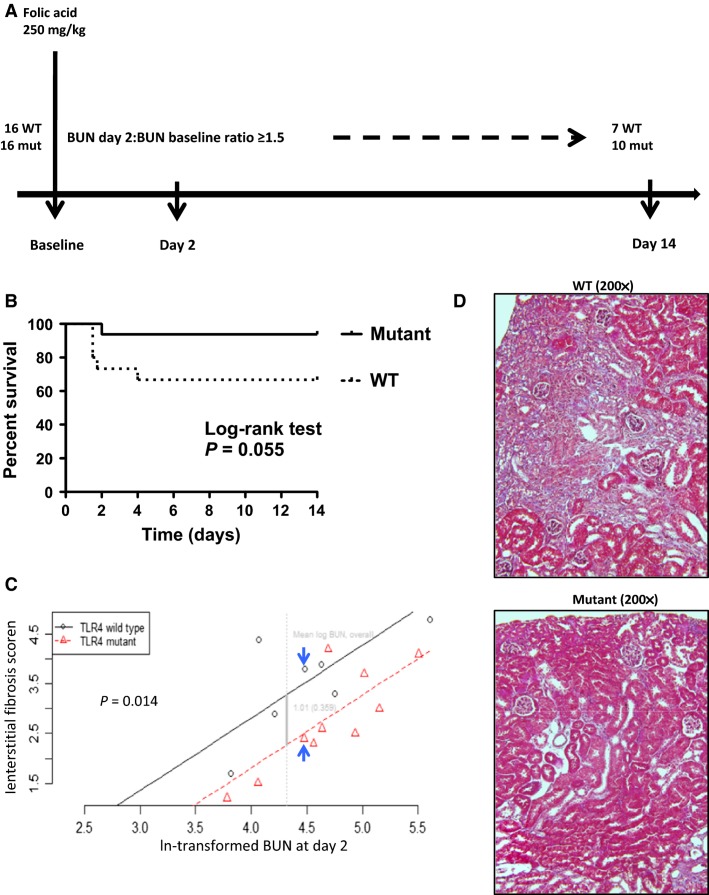
Effect of TLR4 deficiency on renal fibrosis following folic acid injection. (A) Schematic view of the folic acid model. (B) Survival curves after folic acid injection during the length of study. (C) Relationship between severity of acute kidney injury, as indicated by day 2 BUN (log-transformed), and severity of interstitial fibrosis at day 14. TLR4 mutant mice exhibit protection from more severe kidney fibrosis relative to the TLR4 wild type among complete cases, adjusting for postinjury (day 2) BUN: the mean decrease in fibrosis score was –1.01 ± 0.359; *P* = 0.014). (D) Masson trichrome staining: TLR4 wild-type (top panel) and TLR4 mutant (bottom panel), comparing mice that were matched (see arrows in panel C) by log-transformed BUN values at day 2.

Nine week old C3H/HeOuJ (TLR4 WT) and C3H/HeJ (TLR4 mutant) mice were administered folic acid (Sigma-Aldrich, St. Louis, MO) intraperitoneally at a dose of 250 mg/kg in vehicle (0.2 mL of 0.3 mmol/L NaHCO3). Two days later, 50 *μ*L of blood was collected via retro-orbital sinus under isoflurane anesthesia, and blood urea nitrogen (BUN) was measured by colorimetric assay (QuantiChrom Urea assay kit DIUR-500, Hayward, CA). All mice that had a BUN increase at day 2 greater than or equal to 1.5-fold over baseline BUN (collected 2 days before folic acid injection) were included in the study. All mice were monitored for 14 days when blood was collected and kidneys harvested under terminal isoflurane anesthesia. Mortality after folic acid injection was recorded throughout the study period. The experimental design is depicted in Figure 1A.

### Progressive CKD model (5/6 nephrectomy without or with angiotensin II infusion)

**Figure 2:**
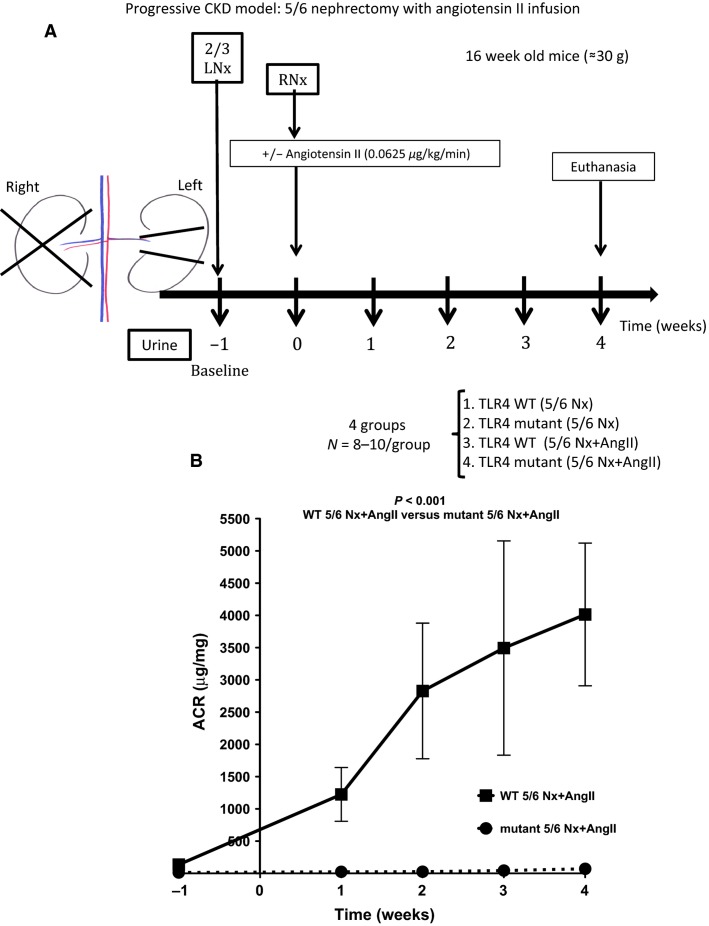
Effect of TLR4 deficiency on albuminuria following 5/6 nephrectomy with angiotensin II infusion. (A) Schematic view of progressive CKD model: mice were subjected to 2/3 left nephrectomy (Nx) on week –1, after baseline urine collection; a week later (zero) mice were subjected to right Nx and a minipump with or without angiotensin II was implanted subcutaneously. Urine was collected weekly and mice were euthanized after 4 weeks. (B) Albuminuria excretion over time (*N* = 8–10/group). ACR, albumin-to-creatinine ratio.

5/6 nephrectomy (5/6Nx) was performed in 16-week-old C3H/HeOuJ (TLR4 WT) and C3H/HeJ (TLR4 mutant) mice in two stages under isoflurane anesthesia. First, the left kidney was decapsulated via left flank incision to avoid ureter and adrenal damage, and the upper and lower poles were resected. Bleeding was controlled with microfibrillar collagen (Avitene; Davol, Warwick, RI). The upper and lower poles were weighed. After 1 week, the entire right kidney was decapsulated and removed via right flank incision. Immediately after each surgical intervention, mice received a single dose of buprenorphine (0.1 mg/kg) diluted in saline (1 mL/25 g), followed by buprenorphine 0.05 mg/kg 18 h after the procedure. Animals with sufficient kidney mass resection [as determined by (removed left kidney weight at week –1)/(removed right kidney weight at week 0) between 0.5 and 0.65] were used for the study; and others, euthanized (Fig. 2A). We found that both C3H/HeOuJ (TLR4 WT) and C3H/HeJ (TLR4 mutant) strains were resistant to CKD progression after 5/6Nx. Since angiotensin II (AngII) overcomes C57BL/6 strain-specific resistance to CKD after 5/6Nx (Leelahavanichkul et al. 2010), we tested the effect of AngII infusion in C3H/HeOuJ (TLR4 WT) and C3H/HeJ (TLR4 mutant) mice subjected to 5/6Nx. AngII at the dose used in C57BL/6 mice (0.75 *μ*g/kg/min) caused 100% mortality within the first 3 days after 5/6Nx+AngII infusion in the strains used in this study: C3H/HeOuJ (TLR4 WT) and C3H/HeJ (TLR4 mutant) mice. In initial studies, we found that a 12-fold lower dose (0.0625 *μ*g/kg/min) of AngII was sufficient to cause progressive kidney disease. This dose of AngII does not have any impact on blood pressure in mice (Zimmerman et al. 2004; Brand et al. 2013). Six mice from each strain, not subjected to any surgery, were euthanized by cardiac puncture under isoflurane anesthesia for blood collection and used as controls.

### Primary renal epithelial cell culture

Renal epithelial cells were isolated from kidneys from TLR4 WT and TLR4 Mutant mice after collagenase digestion (Collagenase D; Roche Diagnostics, Indianapolis, IN). The cells were platted on collagen-coated 24-well plates in Dulbecco’s modified Eagle’s medium (DMEM) supplemented with 10% fetal bovine serum (FBS), 2 mmol/L glutamine, 100 IU/mL penicillin, and 100 *μ*g/mL streptomycin at 37°C in a 5% CO2-humidified atmosphere. After 5 days cells were incubated for 20 h with LPS (0, 10, 50, 100 ng/mL) (Sigma-Aldrich). Supernatant IL-6 was measured by ELISA (R&D Systems, Minneapolis, MN) and normalized by cellular protein content (BCA Protein Assay Kit; Pierce, Rockford, IL). Total RNA was extracted from cells using TRIzol Reagent and purified further with the PureLink RNA Mini Kit after DNase treatment. RNA (2 *μ*g) was reverse-transcribed using TaqMan Reverse Transcriptase Reagents Kit. Real-time qPCR assays were performed with a StepOne Real-Time PCR System (Applied Biosystems, Foster City, CA), with 40 ng of cDNA per reaction. The relative levels of gene expression were measured by the comparative CT (ΔΔCT) method (Schmittgen and Livak 2008) with mouse GAPDH used as a reference gene. All gene expression results were analyzed using 2-ΔΔCT formula and presented as normalized fold changes, compared to control (0 ng/mL LPS control). The TaqMan Gene Expression assays used were as follows: IL-6, IL-1*β*, NLRP3, and GAPDH (Life Technologies ID numbers Mm00446190_m1, Mm00434228_m1, Mm00840904_m1, and Mm03302249_g1). Experiments on primary epithelial cells were performed twice.

### Drug administration

Folic acid (Sigma-Aldrich) was injected once intraperitoneally at a dose of 250 mg/kg in vehicle (0.2 mL of 0.3 mmol/L NaHCO3).

AngII (Val5-AngII) 0.0625 *μ*g/kg/min (Sigma-Aldrich), diluted in sterile water, or vehicle (sterile water) was continuously infused by subcutaneous osmotic mini-pump (Alzet model 1004, Cupertino, CA).

### Blood and urine measurements

Spot urine samples were collected before (baseline), and at 1, 2, 3, and 4 weeks after 5/6Nx (without or with AngII). At 4 weeks, blood was collected by cardiac puncture under isoflurane anesthesia and mice were euthanized. In the folic acid model mice were euthanized 14 days after injection. The remnant kidney (5/6 Nx) and two kidneys (folic acid model) were harvested and fixed in 10% formalin. Serum creatinine was measured by HPLC (Yuen et al. 2004). Urine albumin-to-creatinine-ratio (ACR) was determined from albumin ELISA (Albuwell M; Exocell, Philadelphia, PA) and urinary creatinine by Jaffe method. Serum TNF-*α*, IL-6 (R&D Systems) and HMGB-1 (Shino-Test Corporation, Kanagawa, Japan) were measured by ELISA.

### Morphologic evaluation of the kidney

Kidney specimens were fixed in 10% formalin, paraffin embedded, and stained with Masson’s trichrome and periodic acid-Schiff (PAS) reagent (Sigma-Aldrich). Histological changes were assessed semi-quantitatively. The degree of glomerular damage was assessed in 10 randomly selected fields at 400× magnification from the degree of mesangial expansion in PAS-stained tissue and scored as follows: 1, <25%; 2, 25–50%; 3, 50–75%; 4, >75%; 5, completely sclerotic glomeruli. Interstitial fibrosis was assessed at 200× magnification on Masson’s trichrome-stained sections using 10 randomly selected fields for each animal and scored by the following criteria: 1, area of damage <25%; 2, 25–50%; 3, 50–75%; and 4, 75-100% (Leelahavanichkul et al. 2010).

### Immunohistochemical analysis of activated caspase-3 and programmed death-1 (PD-1) in spleen

Immunohistochemical staining of 4 *μ*m paraffin sections was performed as described previously (Leelahavanichkul et al. 2011), using anti-activated caspase-3 antibody (Cell Signaling Technology, Beverly, MA) and anti-PD-1 (Biolegend, San Diego, CA). The number of positive stained cells in each section was determined from the mean of 10 randomly selected, nonoverlapping 400× fields.

### Statistical analysis

Differences in parameter values between the groups were examined for statistical significance by ANOVA, with subsequent post hoc analysis using Newman–Keuls Multiple Comparison Test. Survival curve with log-rank test was performed for mortality assessment in the folic acid model. Spearman correlation was performed between day 2 log-transformed BUN and interstitial fibrosis score at day 14 in the folic acid model. Because the data at day 14 after folic acid injection may have been influenced by dropouts (deaths), findings were analyzed under a missing completely at random (MCAR) assumption (Little 1988), and fibrosis scores at day 14 were analyzed for each individual mouse taking in consideration its BUN at day 2 (acute injury). Data are expressed as mean ± SEM. Wilcoxon test was used for differences on responses on primary epithelial cells experiments. Analyses were conducted using Prism 5 (GraphPad, San Diego, CA).

## Results

### TLR4 mutant mice are protected from renal fibrosis after folic acid injection

We first tested the role of TLR4 on renal fibrosis using a folic acid model where mice develop tubulointerstitial damage and fibrosis following recovery from folic acid-induced tubular necrosis and acute renal injury. In this relatively simple mouse model mice develop renal damage without a need for angiotensin II infusion. We compared TLR4 WT and mutant mice after folic acid injection. Sixteen TLR4 WT and mutant mice in each group were subjected to intraperitoneous folic acid injection (250 mg/kg of body weight). Immediately after folic acid injection, one mouse in the WT group died of unknown cause. Within the first 2 days after folic acid injection, five mice died out of the remaining 15 in the TLR4 WT group, while one mouse died out of 16 in the TLR4 mutant group (*P* = 0.055; Fig. 1B). As previously described (Doi et al. 2008), BUN at day 2 after folic acid injection significantly correlates with renal interstitial fibrosis at day 14 (*P* = 0.01, *R*^2^ = 0.36); log-transformed BUN at day 2 correlated slightly better (*P* < 0.01, *R*^2^ = 0.40). Mice that had a day 2 BUN/baseline BUN ratio equal to or higher than 1.5 were included in the 14-day study (seven mice in the TLR4 WT group and 10 in the TLR4 mutant group). After adjustment for log-BUN at day 2 (severity of AKI for each mouse), TLR4 mutant mice were protected from interstitial fibrosis (*P* = 0.014) at day 14 in comparison to WT mice (Fig. 1C). Typical histology is shown in Figure 1D, mice matched for day 2 BUN (Fig. 1D).

### TLR4 mutant mice, subjected to 5/6 nephrectomy with concomitant angiotensin II infusion, do not develop albuminuria

We next tested the role of TLR4 in a more complete and clinically relevant model of progressive CKD, 5/6 nephrectomy with angiotensin infusion (Leelahavanichkul et al. 2010). Four weeks after 5/6Nx alone, neither TLR4 WT nor TLR4 mutant mice developed albuminuria (albumin-to-creatinine ratio = 114.1 ± 25.74 and 113.4 ± 24.58 *μ*g/mg, respectively). After 5/6Nx with concomitant infusion of low-dose AngII, TLR4 WT mice developed significant progressive albuminuria starting at week 1, whereas TLR4 mutant mice were completely protected (Fig. 2B).

### TLR4 mutant mice, subjected to 5/6 nephrectomy with concomitant angiotensin II infusion, do not develop progressive kidney dysfunction

**Figure 3:**
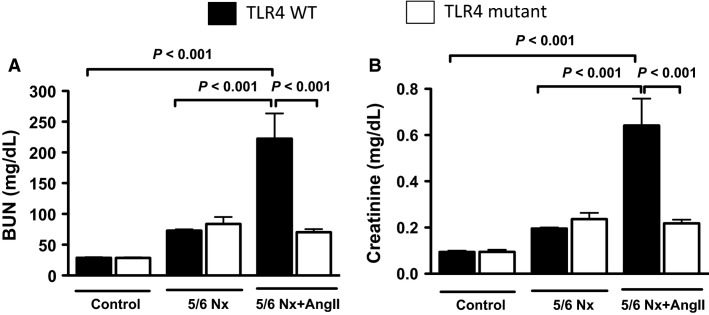
Effect of TLR4 deficiency on kidney function in a progressive CKD model. (A) Serum BUN and (B) serum creatinine at 4 weeks: Control (mice not subjected to any procedure), 5/6Nx alone, and 5/6Nx with angiotensin II infusion (5/6Nx+AngII) (*N* = 6–10/group).

Four weeks after 5/6Nx alone, both TLR4 WT and mutant mice had serum levels of BUN and creatinine that were not statistically different from control animals (mice not subjected to any surgery) (Fig. 3). Four weeks after 5/6Nx with concomitant angiotensin II infusion, TLR4 WT mice had significant increases in both serum BUN and creatinine, whereas TLR4 mutant mice were protected (Fig. 3). Because mice subjected to 5/6 nephrectomy alone (without AngII) did not develop progressive CKD, they were used as controls for the 5/6Nx + AngII group (designated control 5/6 nephrectomy group).

### TLR4 mutant mice, subjected to 5/6 nephrectomy with angiotensin II infusion, do not develop glomerulosclerosis or interstitial fibrosis

**Figure 4:**
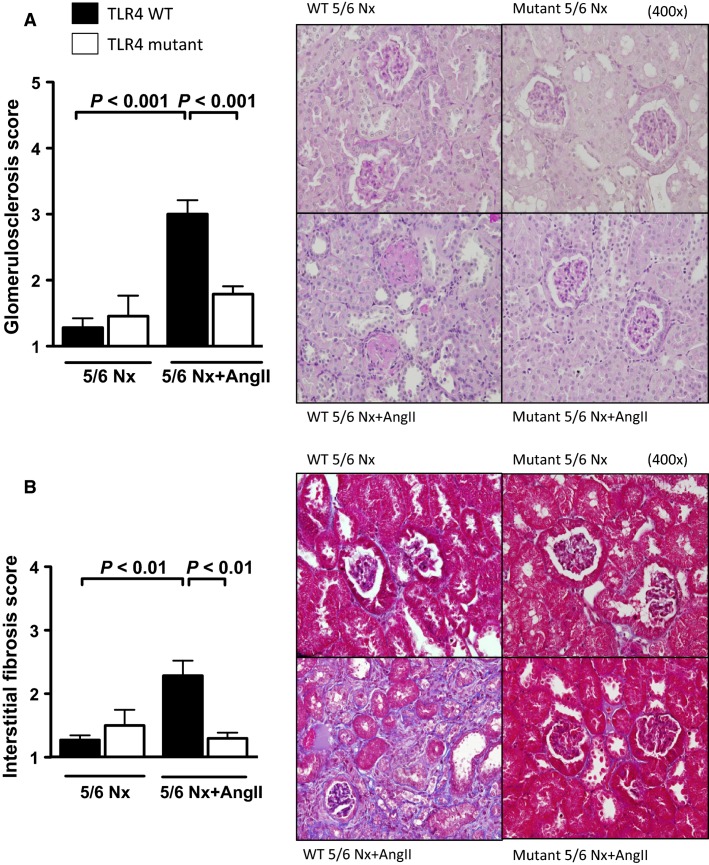
Effect of TLR4 deficiency on renal histological scores in a progressive CKD model. Glomerulosclerosis (PAS stain, A) and interstitial fibrosis (Masson Trichrome stain, B) scores 4 weeks after 5/6Nx+AngII (*N* = 6–10/group).

In addition to increased albuminuria and loss of kidney function, TLR4 WT mice also developed significant kidney histological damage at 4 weeks after 5/6Nx+AngII, as assessed by increased glomerulosclerosis (Fig. 4A) and interstitial fibrosis (Fig. 4B) scores. Sclerotic glomeruli and areas of interstitial fibrosis were rarely found on kidney sections from TLR4 mutant mice, with histological scores similar to mice subjected to 5/6 nephrectomy alone (without AngII infusion).

### TLR4 mutant mice, subjected to 5/6 nephrectomy with angiotensin II infusion, do not develop increases in splenocyte apoptosis, splenic PD-1-positive staining, or increased serum HMGB-1 levels

**Figure 5:**
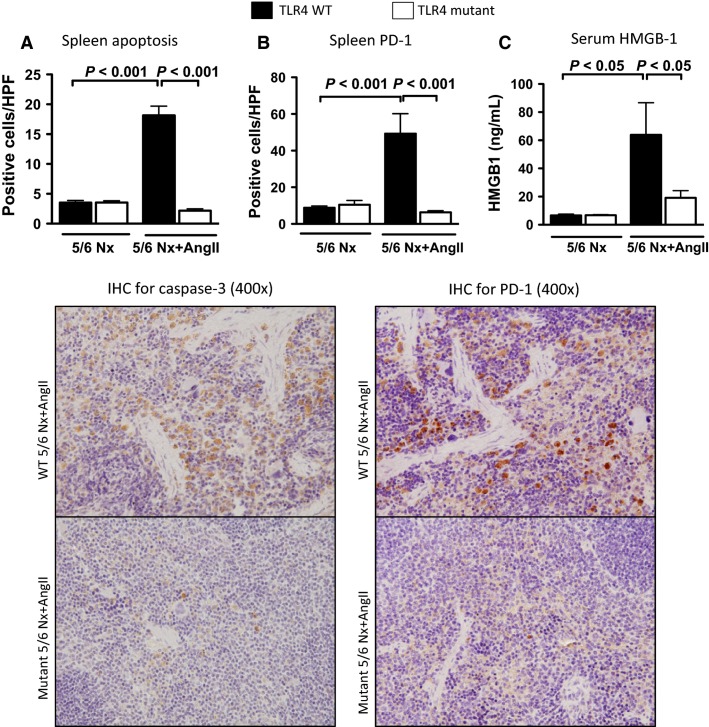
Effect of TLR4 deficiency on splenocyte apoptosis, splenic PD-1 expression, and serum HMGB-1. (A) Spleen apoptosis measured by caspase-3 positive staining (*N* = 6–8/group). (B) Splenic PD-1 cell staining (*N* = 4/group). (C) Serum HMGB-1 levels (*N* = 7–8/group) in mice 4 weeks after 5/6Nx+AngII.

Four weeks after 5/6 nephrectomy with angiotensin II infusion, TLR4 WT mice had significant splenocyte apoptosis (as analyzed by immunohistochemistry for caspase-3), whereas TLR4 mutant mice had very few apoptotic splenocytes (Fig. 5A), similar to mice subjected to 5/6Nx alone. Programmed Death-1 receptor (PD-1) is a cell surface protein molecule expressed on activated T cells and other immune cells that negatively regulate immune responses (Blank and Mackensen 2007). TLR4 WT mice subjected to 5/6Nx+AngII had significantly increased expression of PD-1 in the spleen, which was dramatically lower in TLR4 mutant mice (Fig. 5B). Serum HMGB-1 was significantly increased in TLR4 WT mice 4 weeks after 5/6Nx+AngII, and unchanged in TLR4 mutant mice (Fig. 5C).

### TLR4 mutant mice, subjected to 5/6 nephrectomy with angiotensin II infusion, do not develop low-grade systemic inflammation

**Figure 6:**
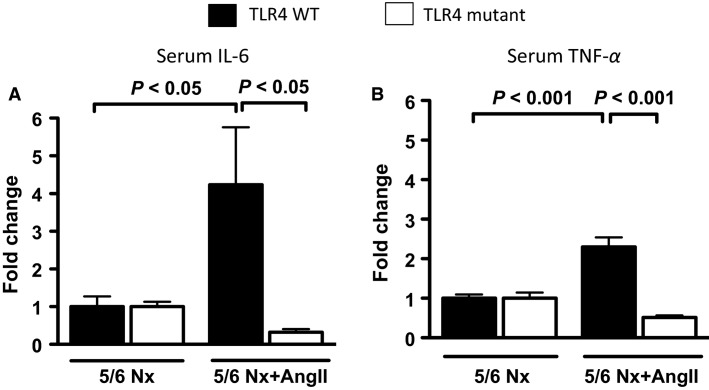
Effect of TLR4 deficiency on systemic inflammation in a progressive CKD model. (A) Serum IL-6 and (B) serum TNF-*α* levels expressed as fold changes over nonprogressive CKD (5/6Nx alone) model (*N* = 7/group).

TLR4 WT mice subjected to 5/6 nephrectomy with angiotensin II infusion also developed increases in systemic inflammation (as assessed by serum IL-6, and TNF-*α*) when compared to mice subjected to 5/6Nx alone (Fig. 6A and B). After 5/6Nx with angiotensin II infusion, IL-6 and TNF-*α* fold change increases over 5/6Nx alone (no progressive CKD) were significantly lower in TLR4 mutant mice than in TLR4 WT mice.

### Effects of TLR4 deficiency on renal epithelial cells

**Figure 7:**
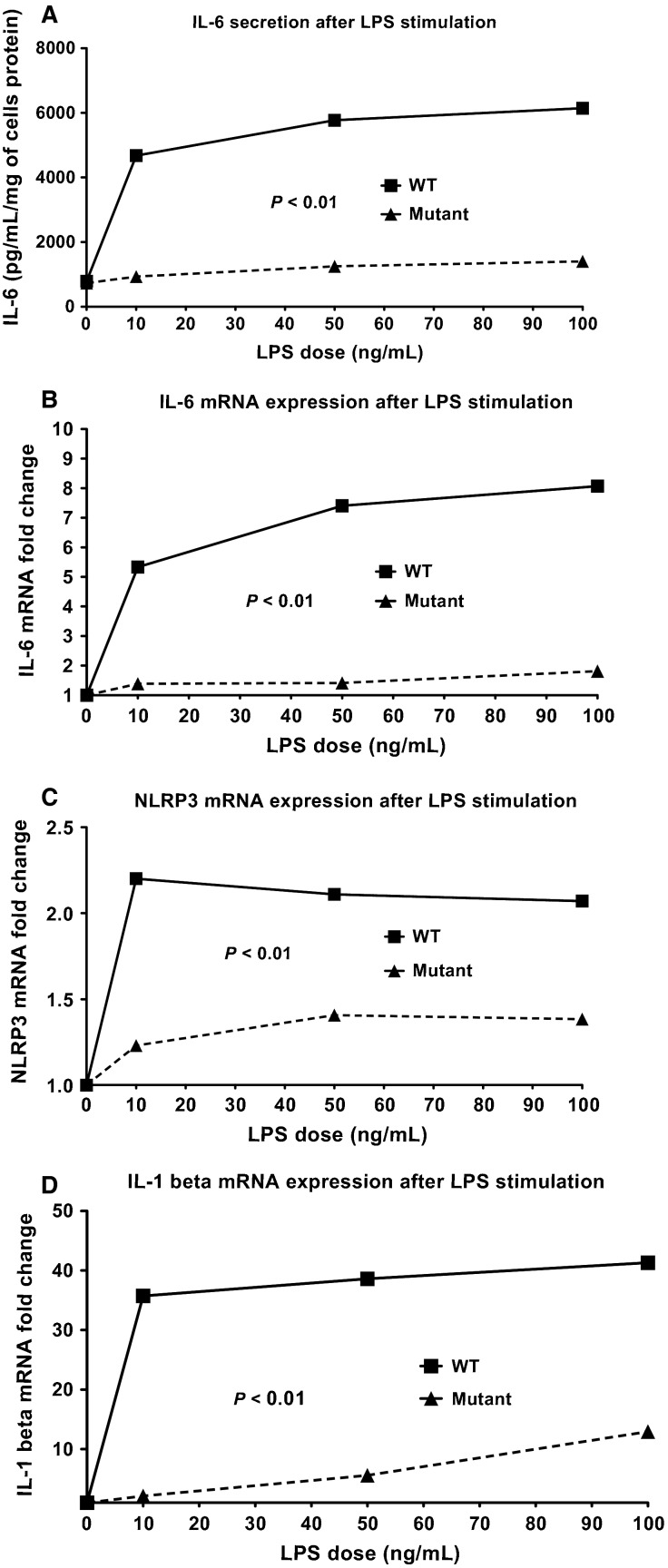
Effect of TLR4 deficiency on primary renal epithelial cell culture. TLR4 WT- and mutant-derived renal epithelial cells were challenged with different concentrations of LPS for 20 h. IL-6 levels in the supernatant (A), and cellular mRNA expression of IL-6 (B), NLRP3 (C) and IL-1*β* (D).

The exact causes of the chronic systemic inflammation during CKD are unknown, but several studies have hypothesized that leakage of gut microbial products into the systemic circulation, due to intestinal microbiome dysbiosis (Vaziri et al. 2012; Anders et al. 2013; Ramezani and Raj 2014) and uremia-related increases in intestinal permeability (Magnusson et al. 1991; de Almeida Duarte et al. 2004; Sun et al. 2009; Vaziri et al. 2012; Wang et al. 2012; Anders et al. 2013; Kubotera et al. 2013; Ramezani and Raj 2014), may cause low-grade endotoxemia during CKD. To test the direct effects of endotoxin (LPS) on renal parenchymal cells, renal epithelial cells isolated from TLR4 WT and TLR4 mutant mice were incubated with LPS. TLR4 WT-derived renal epithelial cells dose-dependently secreted significant amounts of IL-6 in the supernatant when challenged with LPS (Fig. 7A), while renal epithelial cells derived from TLR4 mutant mice secreted low levels of IL-6 at all LPS doses (Fig. 7A), and had lower levels of IL-6 mRNA expression (Fig. 7B). We also examined mRNA expression of inflammasome-related genes NLRP3 and IL-1*β*. LPS up-regulated mRNA expression of both NLRP3 (Fig. 7C) and IL-1*β* (Fig. 7D) in TLR4 WT renal epithelial cells. NLRP3 and IL-1*β* mRNA expression were significantly lower in TLR4 mutant renal epithelial cells stimulated by LPS.

## Discussion

In this study we show that, in vivo, TLR4 mutant mice are protected from renal fibrosis following folic acid injection, and also from progressive CKD in a low-dose angiotensin II infusion (AngII) + 5/6 nephrectomy model. In vitro, TLR4 is required for induction of inflammation and inflammasome activation in primary renal epithelial cells stimulated by LPS. In the progressive CKD model (5/6Nx+AngII), TLR4 mutant mice were protected from CKD-associated low-grade systemic inflammation, spleen apoptosis, and increased splenic expression of the co-inhibitor receptor PD-1, a marker of T-cell exhaustion and immune dysfunction (Chang et al. 2014). Our observations suggest that TLR4 contributes to CKD progression and may also participate in a dysregulated immune response associated with CKD.

### TLR4 mutant mice are protected from renal fibrosis in a model that does not require Angiotensin II infusion

We found that TLR4 mutant mice are protected from interstitial fibrosis in the folic acid model. Others have shown that, 7 days after unilateral ureteral obstruction (UUO), TLR4 knock-out mice have decreased renal fibrosis of the obstructed kidney and decreased proteinuria in urine collected from the unobstructed kidney in comparison to WT mice (Braga et al. 2012). Similarly, in an adenine-induced tubulointerstitial fibrosis model, TLR4 knock-out mice are protected from interstitial fibrosis and increases in serum creatinine (Correa-Costa et al. 2011). These data, together with ours, suggest that TLR4 may play an important role in kidney scarring and fibrosis through pathways independent of extrinsic angiotensin II infusion. The exact mechanism by which TLR4 participates on renal fibrogenesis is yet really unclear. It has been suggested that TLR4 promotes renal fibrosis by modulating the susceptibility of renal tubular epithelial cells and/or myofibroblasts to TGF-β (Pulskens et al. 2010), shifting the balance between M1:M2 macrophage responses (Braga et al. 2012), and fibroblast accumulation (Campbell et al. 2011) during renal fibrogenesis. The loss of TLR4 from proximal tubular epithelial cells in a rat 5/6 nephrectomy model at a late time point (end of study) (Kacso et al. 2015) suggests that if tubule TLR4 contributes the CKD progression, the action is early, perhaps similar to the role of TLR4 in ischemia/reperfusion (Wu et al. 2007) or cisplatin (Zhang et al. 2008) acute kidney injury models.

### TLR4 mutant mice do not develop albuminuria or loss of kidney function in a progressive CKD model

One of the most striking results of our study is that TLR4 mice do not develop albuminuria following 5/6Nx with angiotensin II infusion. Similar findings were seen by Ma et al. in a streptozotocin-induced diabetic nephropathy model, however the degree of albuminuria inhibition was only about 50% vs. 99% in our model (Ma et al. 2014). In our progressive CKD model, we found that 4 weeks after 5/6 nephrectomy with angiotensin II infusion, TLR4 mutant mice were also protected from kidney histological damage and decreased kidney function in comparison to WT mice. The role of TLR4 on diabetic nephropathy has been extensively explored in preclinical studies but, to our knowledge, our work is the first to describe the impact of TLR4 on CKD progression in a model that resembles nondiabetic human disease with characteristics that include progressive albuminuria, loss of kidney function, glomerulosclerosis, and interstitial fibrosis.

### TLR4 mutant mice are protected from low-grade inflammation and immune dysfunction associated with CKD

Consistent with human CKD (Stenvinkel et al. 1999; Silverstein 2009; Tbahriti et al. 2013; Yilmaz et al. 2014), low-grade inflammation was present in mice that developed progressive CKD (TLR4 WT), with increased systemic levels of pro-inflammatory cytokines (HMGB-1, IL-6 and TNF-*α*). In humans, serum IL-6, and TNF-*α* are inversely associated with kidney function, and positively with albuminuria (Gupta et al. 2012). TLR4 WT mice subjected to 5/6Nx+AngII also had increased lymphocyte dysfunction as assessed by the increased amount of spleen apoptosis (caspase-3 activation), and increased expression of the immune inhibitory receptor PD-1 in the spleen, whereas TLR4 mutant mice were protected. Because PD-1 induction can suppress innate immune responses to bacterial infections (Yao et al. 2009; McKay et al. 2015) the TLR4-dependent increase in PD-1 during CKD progression may be one mechanism by which CKD patients become more susceptible to bacterial infections (Naqvi and Collins 2006; Dalrymple and Go 2008). Although inflammation and immunosuppression are seemingly divergent aspects of the innate immune response, they may be regulated by TLR4 at multiple checkpoints, analogous to NFκB participating in not only the onset of TLR-mediated inflammation, but also during the resolution of inflammation (Ghosh and Hayden 2008).

While TLR4 has well-known roles in the immune system, the presence of TLR4 in parenchymal renal cells has not been fully examined. Renal epithelial cells express TLR4 (Wolfs et al. 2002; El-Achkar et al. 2006; Wu et al. 2007; Xiao et al. 2015) and therefore, can be important targets of several TLR4 ligands in the kidney. LPS is a well-established and studied TLR4 ligand, and is found in increased levels among CKD patients (McIntyre et al. 2011). In vitro, LPS directly upregulates genes associated with inflammation and inflammasome activation in renal epithelial cells via TLR4. Other known and unknown ligands that interact directly or indirectly with TLR4 may also contribute to CKD progression via local renal pro-inflammatory signals, including HMGB-1 and angiotensin II (Doi et al. 2014; Lv et al. 2015; Nair et al. 2015).

In summary, we conclude that TLR4 may have an important role in renal interstitial fibrosis and CKD progression. Further studies are warranted to analyze the role of TLR4-inflammasome axis in different models of renal disease and whether similar pathways operate in human CKD.
